# Comparative analysis of blood protein fractions in two mediterranean farmed fish: *Dicentrarchus labrax* and *Sparus aurata*

**DOI:** 10.1186/s12917-024-04182-w

**Published:** 2024-07-18

**Authors:** Sébastien Alfonso, Eleonora Fiocchi, Lola Toomey, Marilena Boscarato, Amedeo Manfrin, Arkadios Dimitroglou, Leonidas Papaharisis, Eleonora Passabi, Annalisa Stefani, Giuseppe Lembo, Pierluigi Carbonara

**Affiliations:** 1 Fondazione COISPA ETS, Bari, Italy; 2 Université Côte d’Azur, CNRS, ECOSEAS, Nice, France; 3 National Reference Laboratory for Fish, Mollusc and Crustacean Diseases, Istituto Zooprofilattico Sperimentale delle Venezie, Legnaro, Italy; 4 Department of Animal Science, Laboratory of Applied Hydrobiology, Agricultural University of Athens, Athens, Greece; 5 Department of Research and Development, AVRAMAR S.A, Paiania, Greece; 6 Laboratory Medicine Service, Istituto Zooprofilattico Sperimentale delle Venezie, Legnaro, Italy

**Keywords:** Aquaculture, European sea bass, Gilt-head bream, Protein fraction, Welfare

## Abstract

Total protein levels in fish are widely used in health and welfare studies, providing a simple and accessible measure. However, the multifaceted role of blood proteins makes it sometimes challenging to link total protein content to specific health issues, while specific protein fractions may offer more precise insights into fish biology and health, particularly in farmed fish species where such data is often lacking. Data were gathered from two experiments involving *Dicentrarchus labrax* and *Sparus aurata*, key species in European marine aquaculture. The aim was (1) to assess how different globulin fractions contribute to total protein content in blood and (2) how these contributions vary across different sampling times in healthy animals. In *D. labrax*, the beta1 globulin fraction emerged as the major contributor (34.16%), followed by albumin and alpha2 globulins (18.24% and 16.41%, respectively). In contrast, pre-albumins and alpha1 fractions had the least contribution (5.49% and 7.71%). *S. aurata* exhibited albumin as the primary contributor (23.39%), followed by beta1 and alpha2 globulins (19.71% and 19.15%, respectively), with gamma and alpha1 fractions contributing the least (5.34% and 8.63%). Notably, the study revealed relatively stable contributions of globulin fractions to total proteins within both species, albeit with minor variations over time, potentially linked to environmental and individual factors. Furthermore, larger fish displayed higher total protein levels. This research underscores the need for further investigation into the diverse factors influencing globulin contributions to total proteins, ultimately enhancing health and welfare monitoring for farmed fish species.

**Supplementary Information:**

The online version contains supplementary material available at 10.1186/s12917-024-04182-w.

## Introduction

Fish production from aquaculture has greatly expanded during the last decades to face the world’s depletion of natural wild resources and the increasing demand for fish products [1]. Managing the health and welfare of farmed fish is also rapidly gaining importance due to both ethics and productivity reasons, making it a topic of great concern for consumers, producers and regulatory authorities [2, 3]. Most animal welfare definitions are typically linked to biological functions and/or feelings [4], but the feeling measures are often debated in fish due to the controversial nature of the concept [5, 6]. Regarding the biological functions-based definition, fish welfare is mainly linked to the physiological status of the organism, including hematological parameters (e.g., hemoglobin, hematocrit), stress indicators (e.g., plasma cortisol, glucose, lactate), as well as blood protein concentrations [7–9].

In this context, the total protein level is a commonly and easily measured blood parameter in fish health and welfare studies [10]. Circulating blood proteins are involved in a wide range of biological functions, including maintaining osmotic pressure, pH regulation, transporting various metabolites and playing an important role in fish humoral immunity [10, 11]. Levels of total proteins may hence provide insights into the nutritional, immune or health status of fishes [10, 12, 13]. Due to the multifaceted roles of the different proteins contained in blood, it can sometimes be challenging to correlate changes in total protein concentration with fish physiology and health. In contrast, the specific protein fractions may provide accurate and valuable insights when assessing fish health and welfare (e.g. [12, 13]). The major protein fractions in fishes include albumin, alpha globulins (alpha1 and 2), beta globulins (beta1 and 2), and gamma globulin. Briefly, albumins serve as transport proteins and participate in regulating blood volume by maintaining oncotic pressure in body fluids [11, 14]. They are often considered the most abundant plasma protein in animals [11, 15]. Then, the alpha and beta globulin fractions are acute-phase proteins and their levels typically depend on inflammation and illness status [12, 13]. Finally, the gamma globulin fraction consists of circulating immunoglobulins and is primarily involved in fish humoral immunity [16].

The quantification of the different protein fractions in blood is overall achieved through serum protein electrophoresis (SPE) and is extensively used for assessing health in mammals, including humans [17]. The automated capillary electrophoresis (CE) provides the means for considerably decreasing the labor component of SPE. Automated data acquisition and analysis allow standardization of interpretations as well as obvious economic benefits [18]. Due to the well-conserved functions of the blood proteins in vertebrates, protein electrophoresis has also been employed for health diagnostics in bird, reptile and amphibian species [12, 17, 19, 20].

In farmed fish species, many studies have reported the concentration of blood proteins as valuable indicators in response to disease, various environmental and/or aquaculture conditions (e.g., Atlantic salmon (*Salmo salar*), European sea bass (*Dicentrarchus labrax*), carp (*Cyprinus carpio*), gilthead sea bream (*Sparus aurata*), Nile tilapia (*Oreochromis niloticus*) or rainbow trout (*Oncorhynchus mykiss*); [13, 21–25]). For instance, in *S. salar*, the albumin level decreased significantly following lice infection [26]. In another example, in *S. aurata*, the concentration of total plasma proteins measured in fish affected by winter syndrome was greater than in healthy fish. This was due to a specific increase in beta2 and gamma globulin levels, while the alpha2 globulin concentration was lower and albumin and alpha1 and beta2 globulins remained unchanged [22]. It is also worth mentioning that the albumin/globulin (A/G) ratio is often used as a reliable indicator for diagnosing health problems, including in fish (e.g., [27–29]). However, data on the specific different protein fractions of fish are scarce in certain farmed fish species (e.g., [13, 22, 23, 27]). This is of particular interest because protein concentration in fish blood may significantly vary both at intra- and interspecific levels (i.e. 0.74 to 7.5 g/dl; [10]) due to many factors (e.g., environment, feeding, diet, health status). For instance, the albumins’ contribution can range from 10 to 50% of the total protein content [11, 15]. Hence, there is a need for data on the concentration and the contribution of the different blood protein fractions in different environmental conditions for farmed fish species of interest to better diagnose and interpret the variations due to health issues [30].

In this study, we collected data from two different experiments performed on healthy individuals of *D. labrax* and *S. aurata*, two of the most important species for European marine aquaculture [1, 31]. We aimed at evaluating (1) how the different globulin fractions contribute to the total protein content in blood and (2) how this contribution may vary over time, across three different sampling times (T0, T1 and T2). In addition, size and species effects were investigated and discussed as potential factors influencing the protein content in blood. This study provides some basic information about the concentrations and contributions of the different protein fractions to the total protein content of these two marine fish farmed species. Ultimately, such information will allow us to enhance the use of these markers for health assessment in aquaculture.

## Materials and methods

**Fig. 1:**
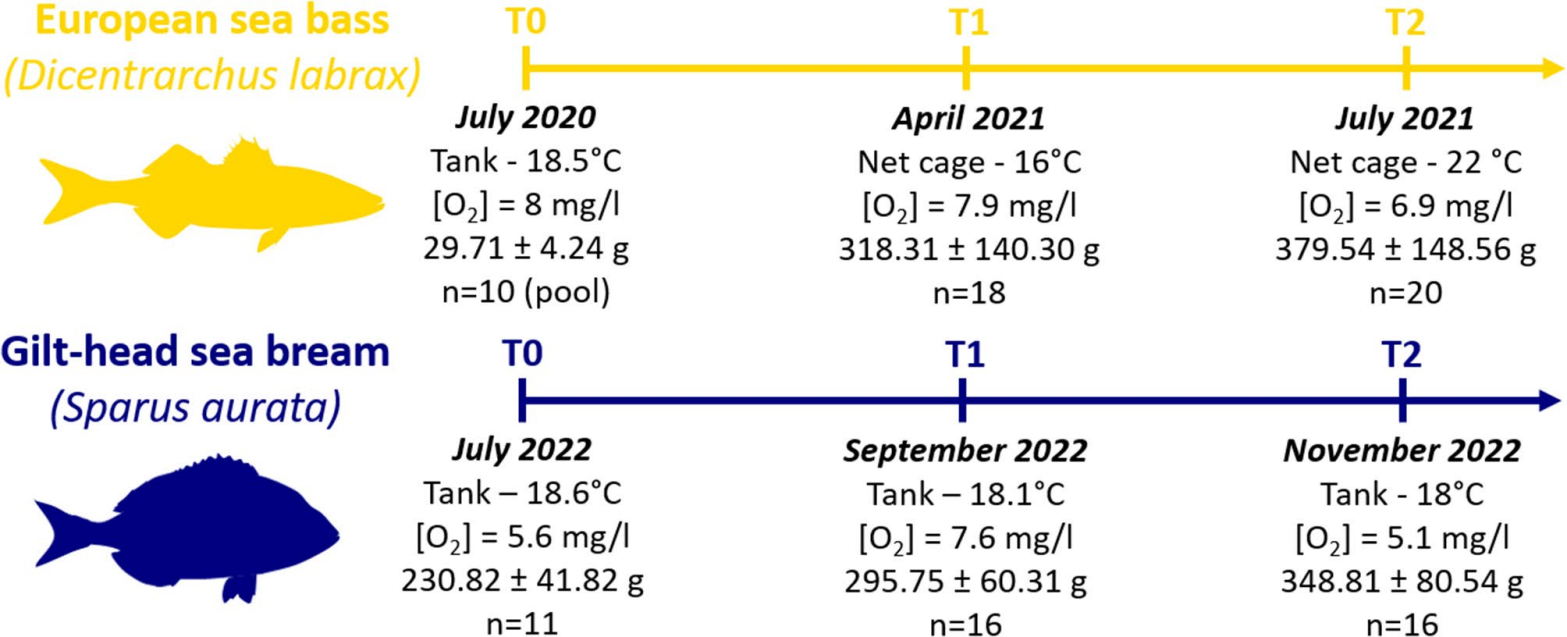
Timeline of the experimental protocol performed with details regarding fish from the two species (European sea bass, *Dicentrarchus labrax*, and gilthead sea bream, *Sparus aurata*) and environmental conditions for each sampling time (T0, T1 and T2)

The experiments involving *D. labrax* and *S. aurata* were carried out in accordance with EU recommendation (Directive 2010/63/EU) and national legislations on the protection of animals used for scientific purposes, under the specific authorization n°98/24,889 (Greece) and n° 488/2021-PR (Italy) for *D. labrax* and *S. aurata*, respectively. The two experiments presented were conducted in different locations, using different rearing systems and environmental conditions (e.g., temperature, stocking density). All details regarding the environmental conditions and dates of sampling for the two experiments (T0, T1 and T2) are presented in the following sections and summarized in Fig. 1.

### Fish rearing

#### Dicentrarchus labrax

The *D. labrax* individuals used originated from the AVRAMAR inland hatchery (Chalkida, Greece) hatched in October 2019. Fish were reared at the hatchery under stable conditions (mean temperature = 18.2 °C, salinity = 30 PSU and oxygen concentration > 8 mg/L) until they were transferred to sea cages at the AVRAMAR farm located in Palairos (Greece) in August 2020 [32]. Before starting the experiment, fish were individually tagged with a unique radio frequency identification (RFID) tag (Biomark, Idaho, USA).

The experimental setup at sea included three net cages, each containing approximately 2200 fish (final stocking density = 9.5 kg/m^3^). Depending on sea temperature and fish size, daily feeding times ranged from two to four times and the fish were fed *ad libitum* using a feed formulated for the specific trial (see all details for the conventional diet in [32] and in Table S1). During the experimental period, the temperature was 20.4 ± 3.9 °C ranging from a minimum of 15.2 °C to a maximum of 28 °C while oxygen concentration averaged 7.2 ± 0.7 mg/L. Temperatures and oxygen concentration at T0, T1 and T2 were 18.5 °C and 8 mg/L, 16 °C and 7.9 mg/L, and 22 °C and 6.9 mg/L respectively. Weekly variations of temperatures and O_2_ are available in Fig. S1. The experiment in *D. labrax* lasted 358 days (~ 12 months).

#### Sparus aurata

The *S. aurata* individuals used were juveniles obtained from the commercial farm REHOMARE (Gallipoli, Italy) and transferred to Fondazione COISPA ETS’s facilities (Bari, Italy) in June 2022. Fish were reared under stable conditions in the facilities (temperature of 18.4 ± 0.2 °C, salinity of 35 PSU and oxygen concentration of 6.4 ± 0.6 mg/L) at a stocking density of ~ 20 kg/m^3^ and a photoperiod of 12 Light:12 Dark (light from 6 am to 6 pm). The fish were reared in a flow-through system with water replacement of ~ 25 L/min and fed commercial feed (Skretting Marine 3 P, Italy) amounting to ~ 1% of their body mass until the start of the experiment. The acclimation period lasted two weeks. Before starting the experiment (T0), fish were individually tagged using frequency identification (RFID) tags (DORSET ID, Trovan, Netherlands).

The experimental setup included two fiberglass tanks, each containing 70 fish (stocking density of 12 kg/m^3^). Fish were fed six to seven days per week, amounting to 1% of their body mass using a commercial fish feed, Active 4.5, 4.5 mm (IRIDA SA, Greece; see Table S2 for details regarding feed composition). The water temperature, water quality parameters and photoperiod specified previously were identical and maintained rather constant during the whole experiment duration. Temperatures and oxygen concentration at T0, T1 and T2 were 18.6 °C and 5.6 mg/L, 18.1 °C and 7.6 mg/L, and 18 °C and 5.1 mg/L respectively. Weekly variations of temperatures and O_2_ are available in Fig. S2. The experiment in *S. aurata* lasted 134 days (~ 4.5 months).

### Experiment protocol and sampling

Fish from the two experiments were sampled at three different sampling times over the experiment (i.e., T0, T1 and T2). Fish from both species were gently caught from the rearing tanks (or sea cages) and slightly anesthetized with a hydroalcoholic clove oil solution (15 ppm, Erbofarmosan, Bari, Italy) for measuring the standard length (cm) and weight (g). Specific growth rate (SGR) and feed conversion ratio (FCR) were estimated between T0 and T2. The SGR was calculated using the following equation: SGR = 100*(ln(W_0_) - ln(W_2_))/ T, where W is the total weight of the fish (g) respectively at T0 (W_0_) and at T2 (W_2_), and T is the number of feeding days. The FCR was calculated as the ratio of the feed ingested (kg of dry weight) per biomass of weight gained (kg). Zootechnical data are presented using cage and individual as replicate for *D. labrax* and *S. aurata* experiments, respectively. Mortalities were recorded daily. Further details regarding fish features from both species and environmental conditions at each sampling time are provided in Fig. 1.

At each of the three sampling times, a subsample of fish was randomly selected for blood samples. The sampling sizes were *n* = 10, *n* = 18 and *n* = 20 for *D. labrax* and *n* = 11, *n* = 16 and *n* = 16 for *S. aurata* at T0, T1 and T2, respectively. At T1, each sample consisted of a pool of six fish because the size of the fish was too low for sampling enough blood. Zootechnical data for sampled fish are presented using individual for both *D. labrax* and *S. aurata* experiments expepted for T0 in D.labrax where pool is used as replicate. For sampling, fish were gently caught from rearing tanks (or sea cages) and immersed in an anesthetic solution (clove oil, 50 and 30 ppm for *D. labrax* and *S. aurata*, respectively) for 2–3 min before proceeding to blood sampling. Blood samples were collected from the caudal vein using a heparinized syringe, and then transferred in a tube with K3EDTA (VACUMED, Torreglia, Italy). Subsequently, the blood was centrifuged at 15,000 × g for 3 min to obtain plasma samples, which were stored at − 20 °C until further analysis, as described below. At each sampling time of the two experiments, sampled fish for blood were then euthanized using an overdose of anaesthetics. Necropsy, anatomo pathological observation of internal organs, skin and gill scraping were performed to check fish for potential parasites. No anatomo pathological lesions or parasites were observed in all fish inspected, ensuring that fish analysed in the study were healthy.

### Analysis of protein composition

Electrophoresis for measuring the protein fractions content in plasma (albumin, alpha1, alpha2, beta1, beta2 and gamma) was carried out using Minicap Flex Piercing system (Sebia, Bagno A Ripoli, Italy) according to the manufacturer’s instructions. The Minicap Flex Piercing is a multitasking automated capillary electrophoresis instrument, equipped with two capillaries that allow two electrophoretic separations to be performed simultaneously, without the need for manipulation.

Serum proteins were separated within silica capillaries based on their electrophoretic mobility and electrosmotic flow under high voltage conditions and in alkaline buffer. Proteins were detected directly during migration by UV absorbance at 200 nm and quantified.

The Minicap system allows all electrophoresis sequences to be performed automatically from the primary tube until the electrophoretic profile is obtained. The instrument is equipped with a Phoresis (Sebia) software program that allows the processing of results. Identification of the fractions was performed automatically and the electrophoretic profiles were analysed on the fly.

### Statistical analyses

Statistical analyses were carried out using the R software version 4.0.4 [33] at the 95% level of significance. Data are presented as mean ± SD (standard deviation).

First, the contribution percentage of each globulin to the total protein content, the concentration of total proteins and the specific concentration of the different globulins (pre-albumin, albumin, alpha1, alpha2, beta1, beta2 and gamma) were evaluated using ANOVA with the sampling time (T0, T1 and T2) as a factor in both *D. labrax* and *S. aurata*. When significant, the ANOVA was followed by Tukey’s honest significant difference (HSD) post-hoc test to determine the specific differences between sampling times.

Then, the effect of body mass on total protein content was evaluated using linear regression, including the species and the interaction between mass and species as additional factors. Since a pool of fish was used for *D. labrax* at T0, we used the average mass of the different fish in the pool at this sampling time. Analyses comparing species were performed using only the data of both species at T1, ensuring that the fish had similar masses for the comparison (318.31 ± 140.30 and 295.75 ± 60.31 g for *D. labrax* and *S. aurata*, respectively; Wilcoxon-Mann-Whitney, *p* > 0.05). Additionally, at T1, the water temperature and oxygen concentration between the two experiments are closer to each other than at T2 (i.e., 16 °C and 18.1 °C vs. 22 °C and 18 °C for temperature; 7.9 mg/L and 7.6 mg/L vs. 6.9 mg/L and 5.1 mg/L for oxygen concentration). Finally, Pearson’s correlation tests were carried out to test the inter-correlations of the different globulins within each species using the whole dataset.

## Results

### Zootechnical data

In the *D. labrax* experiment, the fish grew from 40.9 ± 0 to 474.8 ± 9.6 g, with a SGR of 0.69 ± 0.01%/day and FCR of 2.06 ± 0.09. The mortality over the 358-day experiment was 22.03 ± 1.30%. For *S. aurata*, the fish grew from 219.7 ± 50.0 to 359.7 ± 76.9 g, with a SGR of 0.46 ± 0.20%/day and a FCR of 2.28 ± 2.1 The mortality over the 134-day experiment was 3.57 ± 1.01% (Table S3). Overall, the mass of fish sampled was representative of the population at the different sapling times for both experiments (see Table S3 for details).

### Contribution and variation of the different globulin fractions to the total protein content in the two species

#### Dicentrarchus labrax

**Fig. 2:**
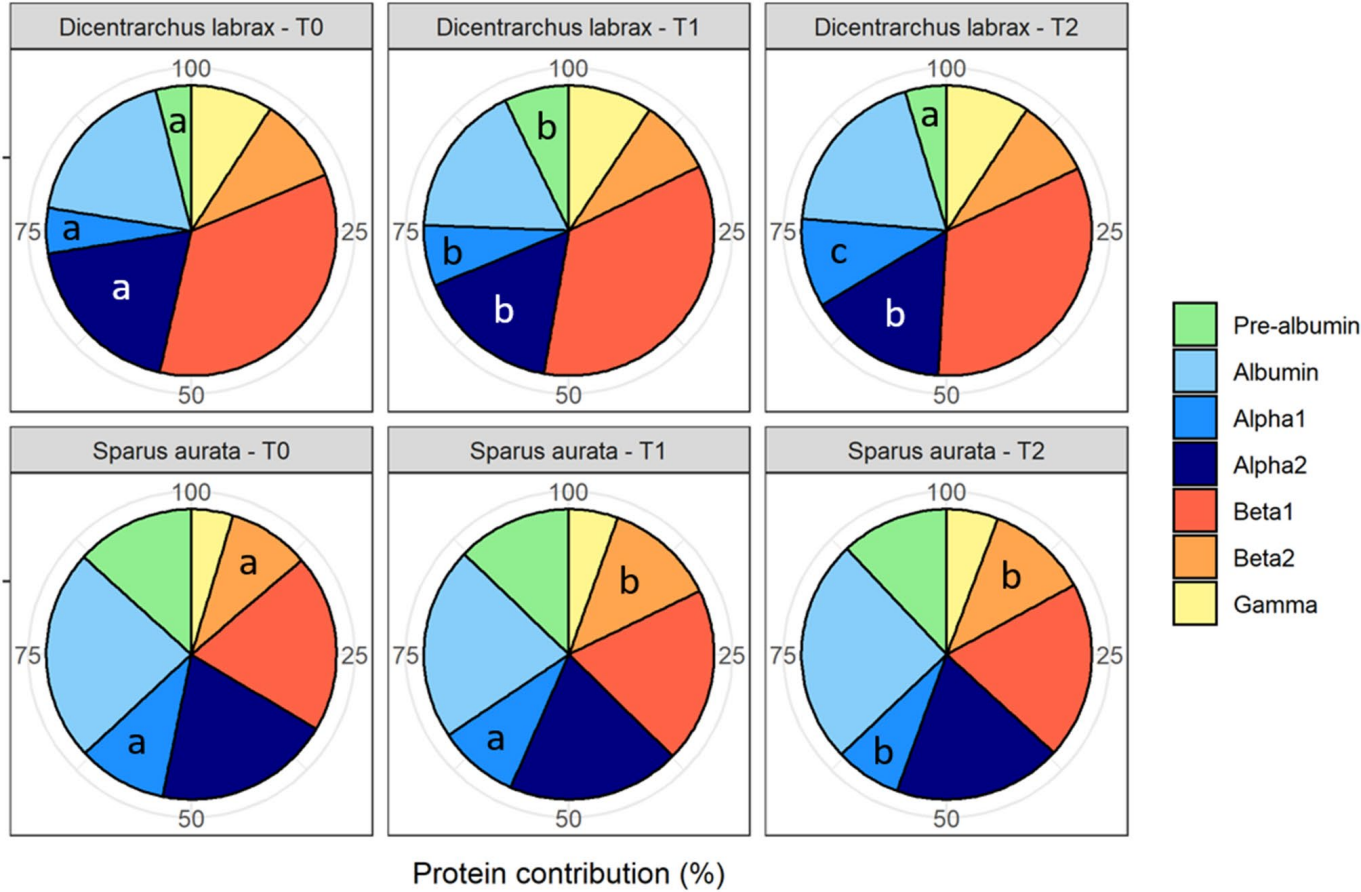
Contribution of the different globulins (pre-albumin in green; albumin in light blue; alpha1 in blue; alpha2 in dark blue; beta1 in red; beta2 in orange; gamma in yellow) to the total protein content (%) in European sea bass (*Dicentrarchus labrax*) and gilthead sea bream (*Sparus aurata*) at the three sampling times (T0, T1 and T2). Sample sizes are *n* = 10, *n* = 18 and *n* = 20 for *D. labrax* and *n* = 11, *n* = 16 and *n* = 16 for *S. aurata* at T0, T1 and T2, respectively. Different letters indicate significant differences within species in the specific fractions between the three sampling times (ANOVA followed by Tukey HSD, *p* < 0.05)

In *D. labrax*, the percentage contribution of different globulin fractions to the total protein content remained generally consistent across the three sampling times for most globulins (albumin, beta1, beta2 and gamma, *p* > 0.05; Fig. 2). However, there were variations over time in the contribution percentage of pre-albumin, alpha1 and alpha2globulins (*p* < 0.05; Fig. 2). Specifically, the contribution of pre-albumin was higher at T1 compared to T0 and T2 while the contribution of alpha1 was higher at T2 than T1 and was higher at T1 than T0, and alpha2 was higher at T0 than T1 and T2 (*p* < 0.05; Fig. 2). Overall, the pre-albumin fraction contributed for 5.49 ± 3.53% (min-max = 0.70–16.50%), the albumin fraction for 18.24 ± 6.94% (min-max = 1.20–30.20%), the alpha1 and alpha2 globulin fractions for 7.71 ± 2.39% (min-max = 3.90–12.10%) and 16.41 ± 2.06% (min-max = 12.40–21.20%), respectively, the beta1 and beta2 globulin fractions for 34.16 ± 5.47% (min-max = 24.10–52.80%) and 8.71 ± 2.70% (min-max = 4.50–15.60%), respectively, and finally the gamma fraction contributed for 9.29 ± 2.07% (min-max = 0.60–12.70%). The highest coefficients of variation (CV) were observed for the pre-albumin (64.37%) and albumin fractions (38.08%), while the lowest CV were observed for alpha2 (12.59%) and beta1 globulin fractions (16.02%) in *D. labrax*.

**Fig. 3:**
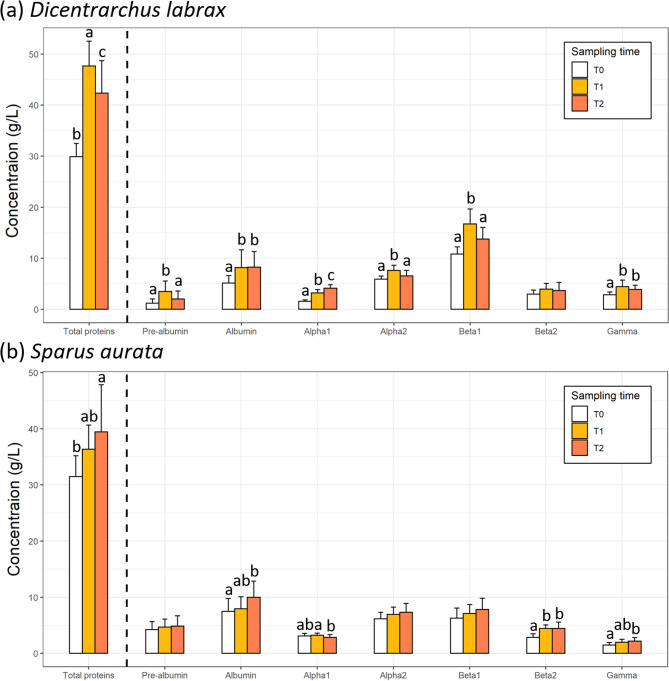
Protein content (g/L, mean ± SD) measured in **(a)** European Sea bass (*Dicentrarchus labrax*) and **(b)** and gilthead sea bream (*Sparus aurata*) at the three sampling times (T0, white; T1, orange and T2, red). Sample sizes are *n* = 10, *n* = 18 and *n* = 20 for *D. labrax* and *n* = 11, *n* = 16 and *n* = 16 for *S. aurata* at T0, T1 and T2, respectively. Different letters indicate significant differences within species between the sampling times (ANOVA followed by Tukey HSD, *p* < 0.05)

In *D. labrax*, the sampling time significantly affected the plasma concentration of total protein and the specific concentrations of pre-albumin, albumin, alpha1, alpha2, beta1 and gamma globulins (*p* < 0.05; Fig. 3a). In more details, the total protein concentration was higher at T1 compared to both T2 and T0, and higher at T2 than T0. Additionally, the concentrations of pre-albumin, alpha2 and beta1 globulins were higher at T1 compared to both T0 and T2. Finally, the concentrations of both albumin and gamma globulins were higher at both T1 and T2 compared to T0 (*p* < 0.05), but there was no difference between T1 and T2 (*p* > 0.05; Fig. 3a). Finally, the concentration of albumin was lower at T0 compared to both T1 and T2, while alpha1 concentration increased continuously from T0 to T2 (*p* < 0.05; Fig. 3a). Overall, the plasma concentration of total proteins in *D. labrax* was 41.75 ± 8.34 g/L (min-max = 25.00–58.00 g/L; CV = 19.98%), in which the specific concentrations were 2.42 ± 1.85 g/L (min-max = 0.19–9.08 g/L; CV = 76.75%) for pre-albumin, 7.57 ± 3.21 g/L (min-max = 0.37–15.10 g/L; CV = 42.36%) for albumin, 3.25 ± 1.14 g/L (1.16–6.12 g/L; CV = 35.24%) and 6.82 ± 1.15 g/L (min-max = 4.59–10.03 g/L; CV = 16.88%) for alpha1 and alpha2 globulins, respectively, 14.27 ± 3.26 g/L (min-max = 8.59–22.39 g/L; CV = 22.82%) and 3.65 ± 1.33 g/L (min-max = 1.74–6.75 g/L; CV = 36.35%) for beta1 and beta2 globulins, respectively, and 3.90 ± 1.13 g/L (min-max = 0.26–6.32 g/L; CV = 28.95%) for gamma globulins (Fig. 3a).

#### Sparus aurata

Similar to *D. labrax*, the percentage contribution of different globulin fractions to the total protein content in *S. aurata* was consistent across the three sampling times for most globulins (pre-albumins, albumin, apha2, beta1 and gamma, *p* > 0.05; Fig. 2). However, there were variations over time in the contribution of alpha1 and beta2 globulin fractions (*p* < 0.05; Fig. 2). Alpha1 contribution was lower at T2 compared to both T1 and T0 while beta2 contribution was lower at T0 compared to both T1 and T2 (*p* < 0.05; Fig. 2). Overall, the pre-albumin fraction contributed for 12.66 ± 3.46% (min-max = 5.00-18.60%), the albumin fraction for 23.39 ± 4.47 ± % (min-max = 12.50–33.00%), the alpha1 and alpha2 globulin fractions for 8.63 ± 1.67% (min-max = 5.70–12.40%) and 19.15 ± 3.40% (min-max = 14.40–28.20%), respectively, the beta1 and beta2 globulin fractions for 19.71 ± 3.58% (min-max = 11.00-27.50%) and 11.13 ± 2.16% (min-max = 5.30–14.30%), respectively, and finally the gamma globulin fraction contributed for 5.34 ± 1.50% (min-max = 2.40–9.90%) to the total protein content. The highest CV were observed for the pre-albumin (27.30%) and gamma globulin fractions (28.13%) while the lowest CV were observed for alpha2 (17.73%) and beta1 globulin fractions (18.17%) in *S. aurata*.

In addition, sampling time had a significant effect on the plasma concentration of total protein, as well as the specific concentrations of albumin, alpha1, beta2 and gamma globulins (*p* < 0.05; Fig. 3b). In more details, the total protein plasma concentration was higher at T2 than T0 while there was no significant difference between T1 and either T0 or T2 (*p* < 0.05). A similar pattern was observed for both albumin and gamma globulin concentrations in *S. aurata*, with higher concentrations at T2 compared to T0, with no significant difference between T1 and either T0 or T2 (*p* < 0.05). Moreover, the alpha1 concentration was lower at T2 compared to T1, but there was no significant difference between T0 and either T1 or T2 (*p* < 0.05). Finally, the beta2 concentration of *S. aurata* was higher at both T1 and T2 compared to T0 (*p* < 0.05; Fig. 3b). Overall, the total protein plasma concentration in *S. aurata* was 36.26 ± 6.71 g/L (min-max = 24.00–51.00 g/L; CV = 18.50%), in which the specific concentrations were 4.63 ± 1.56 g/L (min-max = 1.60–8.20 g/L; CV = 33.78%) for pre-albumin, 8.57 ± 2.65 g/L (min-max = 3.90–15.60 g/L; CV = 30.95%) for albumin, 3.05 ± 0.46 g/L (2.00–4.00 g/L; CV = 15.17%) and 6.87 ± 1.43 g/L (min-max = 4.70–10.20 g/L; CV = 20.73%) for alpha1 and alpha2 globulins, respectively, 7.16 ± 1.86 g/L (min-max = 3.90–11.60 g/L; CV = 26.02%) and 4.04 ± 1.08 g/L (min-max = 1.60–6.10 g/L; CV = 26.78%) for beta1 and beta2 globulins, respectively, 1.93 ± 0.61 g/L (min-max = 0.80–3.30 g/L; CV = 31.85%) for gamma globulin (Fig. 3b).

### Size and species effects on the total protein content and specific globulins

#### Size effect

**Fig. 4:**
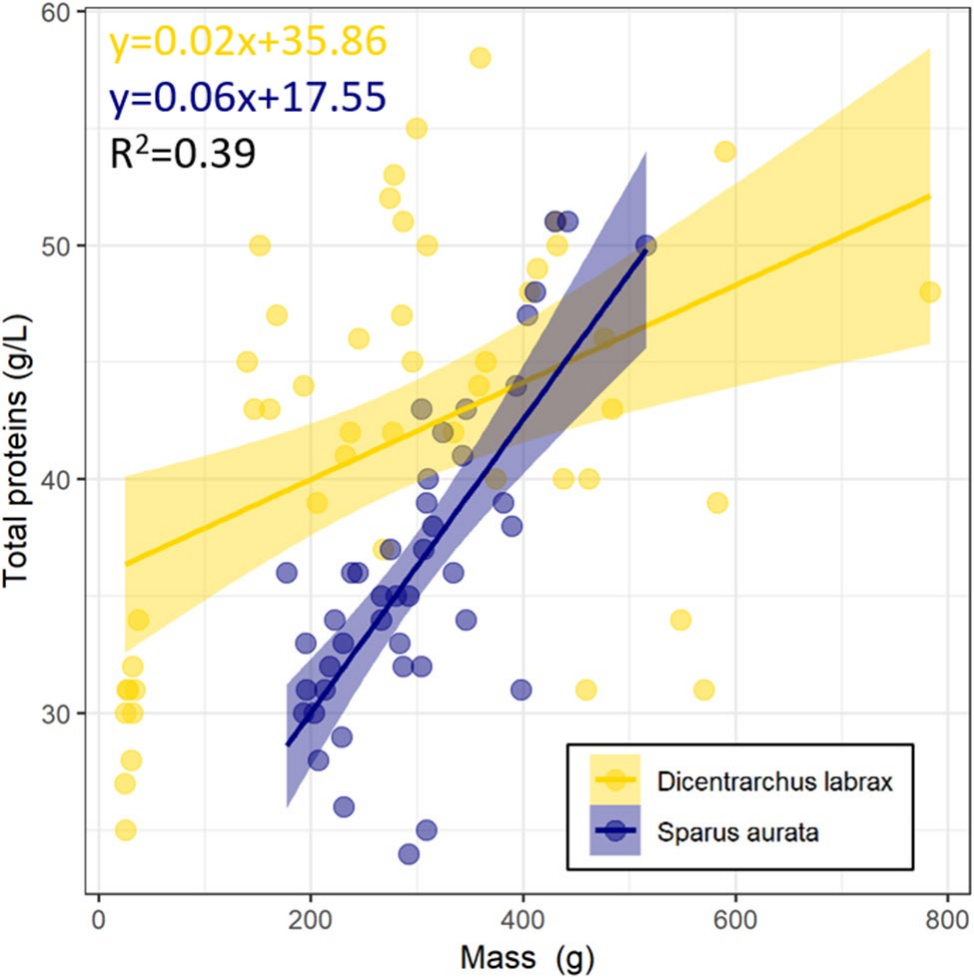
Linear regression between the total blood protein content (g/L) and mass (g) in European sea bass (*Dicentrarchus labrax*, *n* = 48, gold) and gilthead sea bream (*Sparus aurata*, *n* = 43, dark blue)

The plasma concentration of total proteins increased with the body mass of the fish regardless of the species investigated (t = 4.176, *p* < 0.001; Table 1; Fig. 4). However, the relationship between total blood protein concentration and body mass was significantly different between species (t=-4.399, *p* < 0.001) and there was also a significant interaction effect between body mass and species (t = 3.145, *p* < 0.01; Table 1).

**Table 1:** Summary of the linear regression between blood total protein content, mass and the two species investigated (*n* = 48 for *D. Labrax* and *n* = 43 for *S. Aurata*)

	Estimate	Std. error	T value	*p*-value
(Intercept)	35.86149	1.67739	21.379	< 0.001 (***)
Mass	0.02076	0.00497	4.176	< 0.001 (***)
Species:*S. aurata*	-18.31321	4.16332	-4.399	< 0.001 (***)
Mass: Species:*S. aurata*	0.04183	0.01330	3.145	0.00227 (**)

#### Species effect

**Fig. 5:**
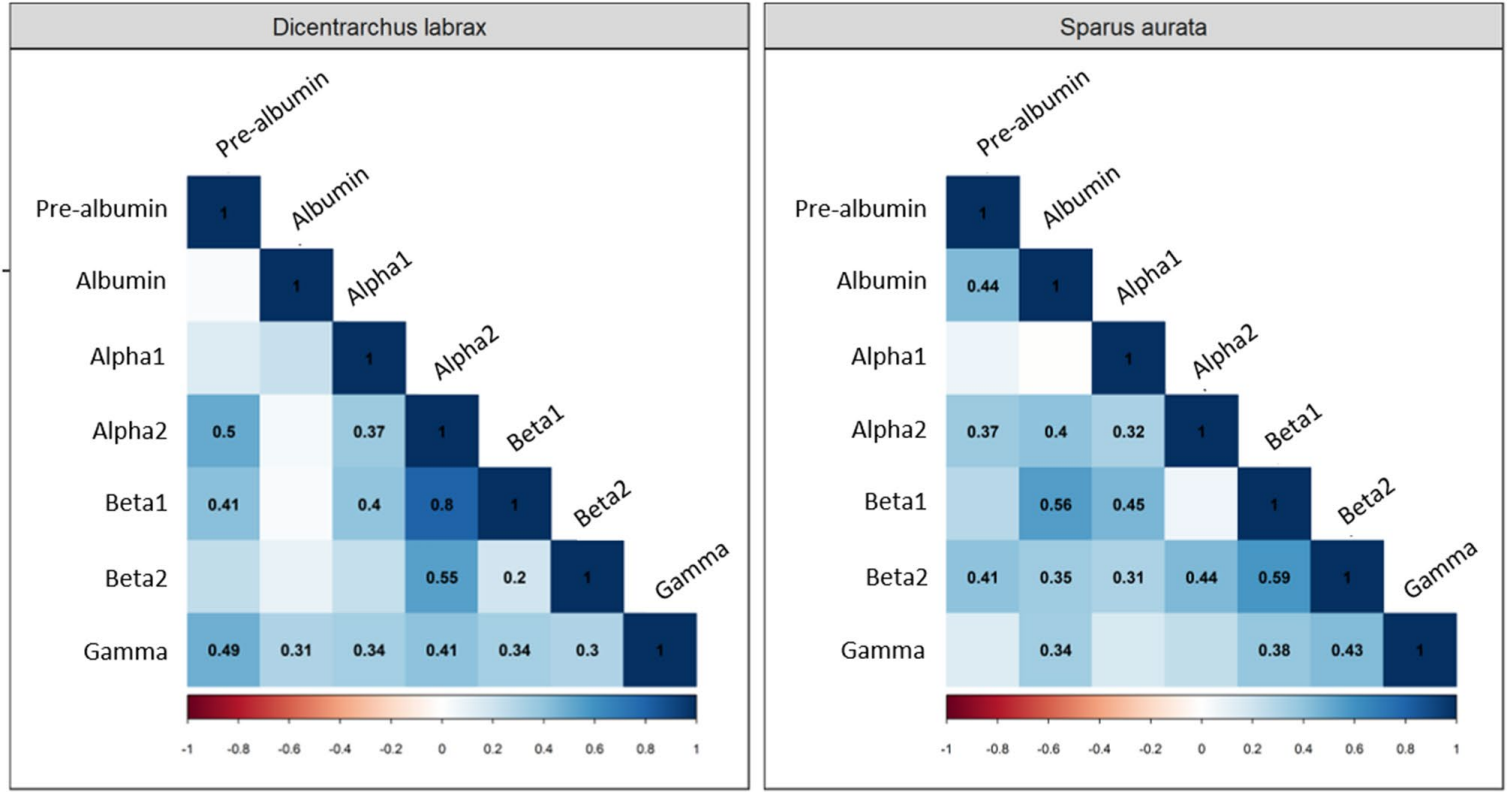
Correlation matrix of the different globulins (pre-albumin; albumin; alpha1; alpha2; beta1; beta2; gamma) in European sea bass (*Dicentrarchus labrax*, *n* = 48) and gilthead sea bream (*Sparus aurata*, *n* = 43). The coefficients of the significant correlations between different globulins are reported in the figure for each species (Pearson’s correlation test, *p* < 0.05)

There were numerous inter-correlations between different globulin concentrations within each species (*p* < 0.05), but only few inter-correlations were present in both species (Fig. 5). Specifically, correlations were observed between the plasma concentrations of pre-albumin and alpha2, albumin and gamma, alpha1 and alpha2, alpha1 and beta1, alpha2 and beta2, beta1 and beta2, beta1 and gamma, and beta2 and gamma in both *D. labrax* and *S. aurata* (Fig. 5).

**Fig. 6:**
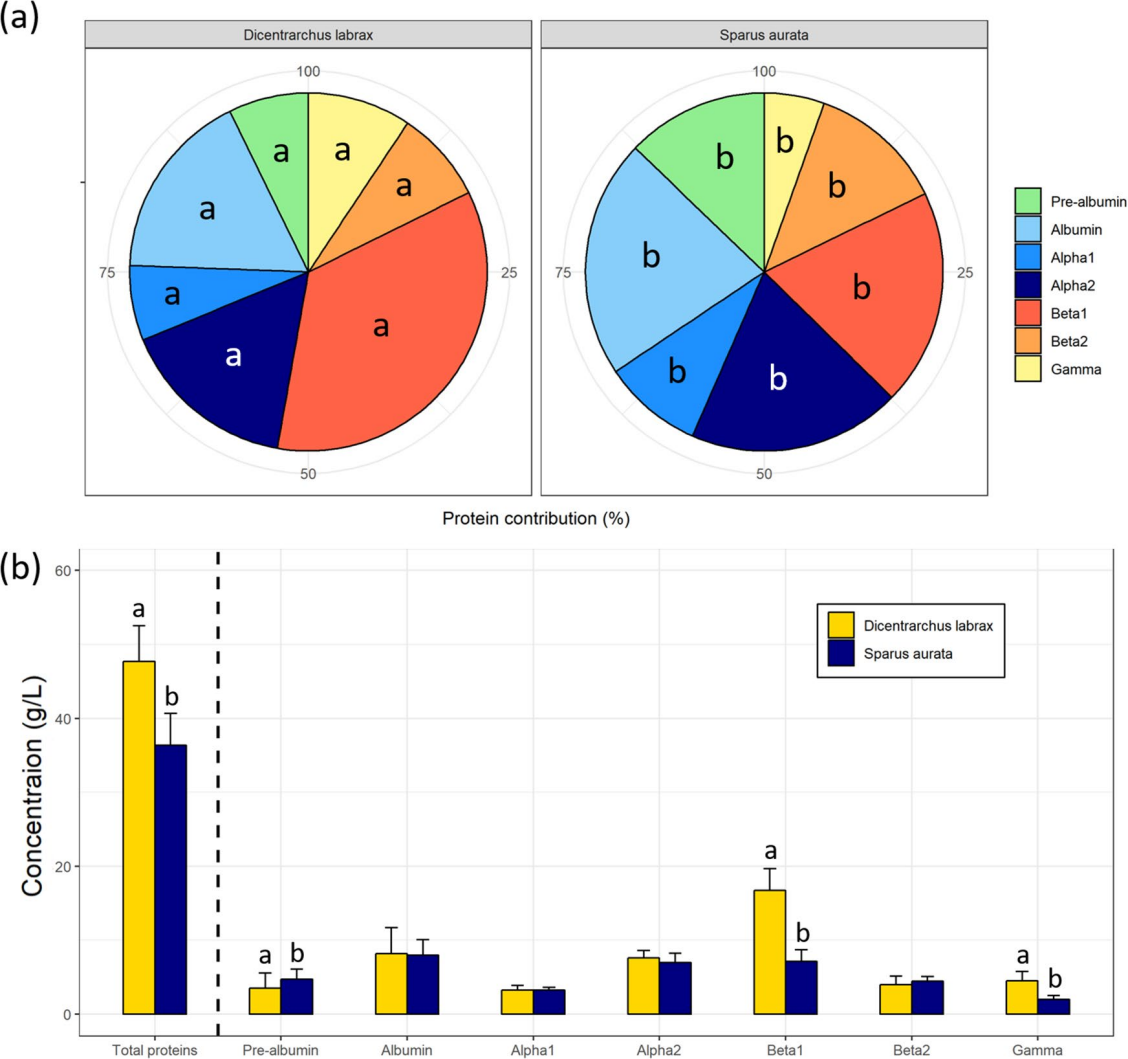
**(a)** Contribution of the different globulins (pre-albumin in green; albumin in light blue; alpha1 in blue; alpha2 in dark blue; beta1 in red; beta2 in orange; gamma in yellow) to the total protein content (%) in European sea bass (*Dicentrarchus labrax*) and gilthead sea bream (*Sparus aurata*) at T1 and **(b)** Protein content (g/Ll; mean ± SD) measured in *D. labrax* (*n* = 18, gold) and *S. aurata* (*n* = 16, dark blue) at T1. Different letters indicate significant differences between the species (T-test, *p* < 0.05)

Overall, the profile of different globulin fractions was significantly different between the two species investigated (*p* < 0.05 for all proteins; Fig. 6). Specifically, pre-albumin, albumin, alpha1, alpha2 and beta2 fractions contributed more to the total protein content in *S. aurata* compared to *D. labrax*, whereas beta1 and gamma fractions contributed less in *S. aurata* (Fig. 6a). When assessing the levels of total blood proteins and specific proteins, there were significant differences between the two species at T1 (Fig. 6b). Specifically, total protein content measured in plasma was higher in *D. labrax* than in *S. aurata*. Additionally, the levels of beta1 and gamma globulins were higher in *D. labrax*, whereas the level of pre-albumin was higher in *S. aurata* (*p* < 0.05; Fig. 6b). However, there were no significant differences in the levels of albumin, alpha1, alpha2 and beta2 globulins between the two species (*p* > 0.05; Fig. 6b).

## Discussion

The levels of blood proteins are of great interest in assessing fish health because they provide valuable insights into how fish are coping with environmental variations and diseases [22, 26, 34]. However, information about the contribution of the different protein fractions to the total protein content and how it varies depending on the environment and individual features in farmed fish species of interest, such as *D. labrax* and *S. aurata*, is overall scarce (e.g. [22, 27, 30, 34]). Such knowledge may benefit the assessment of fish health in aquaculture. In this study, we provide data on how the different globulin fractions contribute to the total protein content and we discuss how this contribution may vary in these two species, as detailed below.

### Zootechnical data

At the end of the trial in the *D. labrax* experiment (358 days), the average weight of fish was 475 g with a SGR of 0.69%/day, supporting relative good performance under the given environmental conditions in sea cages [32, 35]. Regarding mortality, we observed an overall mortality rate of 22%, consistent with the expected mortality rate of 20% throughout the full grow-out stage for *D. labrax* [32, 36]. In the *S. aurata* experiment (134 days), the fish also exhibited growth consistent with species expectations under comparable experimental rearing conditions (i.e., temperature, salinity, fish size) [37], achieving an average weight of 360 g and a SGR of 0.49%/day. Mortality in *S. aurata* was only 3.6%, which aligns with rearing in constant and controlled indoor facilities [37]. Overall, the zootechnical data from both experiments support good fish performance and conditions, as also confirmed by the lack of anatomo pathological lesions or parasites in the fish sampled.

### Contribution of globulin fractions to total protein content and variation of protein concentration in the two species

The concentration of total proteins measured in the plasma of both species fell within the range usually observed in fishes [11], including the studied species [22, 25, 27, 34, 38–41]. Overall, the results of the study show that the contributions of the different globulin fractions to total proteins remain relatively stable within the two species, although some variations can be observed over time. Moreover, the contribution of the different globulins differed significantly between the two species investigated.

In more detail, in *D. labrax*, the percentage contribution of globulins to total protein content was consistent for the majority of globulins (albumin, beta1, beta2 and gamma) across the three sampling times. However, variations over time were observed in the contribution proportions of pre-albumins, alpha1 and alpha2 globulins. In *S. aurata*, a similar trend of low variation over time was observed, but the protein fractions exhibiting variability over time differed. While the percentage contribution of different globulins to total protein content was overall consistent across the three sampling times for most globulins (pre-albumin, albumin, alpha2, beta1 and gamma), there was some variation over time in the contribution of the alpha1 and beta2 globulin fractions to total protein content. The observed variations in globulin fractions over time may be partly attributed to environmental changes, such as stocking density or water quality (e.g., temperature) [42–48]. Serum proteins are involved in a wide range of physiological functions and changes in fish protein fractions have been previously utilized as indicators of environmental stress response [42]. In this study, *D. labrax* individuals experienced a range of temperatures, from 16 °C (T1) to 22 °C (T2), across the three sampling times. Water temperature fluctuations have been previously associated with changes in serum albumin and globulin levels in several species ([34] and references therein; but see the lack of albumin concentration changes in [22]). In this study, changes in *D. labrax* globulin fractions and albumin concentrations were observed in congruence with temperature differences between sampling times. It is, however, worth mentioning that the levels of different blood proteins are not only influenced by the water temperature at the time of sampling but may also be influenced by temperature fluctuations in the preceding days. Although variations in temperature were negligible in the *S. aurata* experiment due to specific rearing conditions, it could be more likely the case in the *D. labrax* experiment where temperature fluctuated more in the days preceding sampling (see Fig S1 and Fig S2 for details). In all cases, the observed changes in contribution and concentration cannot be solely attributed to the temperature recorded on the day of sampling, as other environmental parameters may also play a role. Given that alpha and beta globulins are involved in various functions, such as lipid and cortisol transport, coagulation and immune response [34], their changes may reflect stress experienced due to environmental variations. While the plasma concentration of some globulins was found to vary across different sampling occasions in both species, *D. labrax* displayed more variation than *S. aurata*. This can be partly attributed to the fact that environmental conditions (i.e., temperature, stocking density) and fish features (e.g., size, gonad maturation) underwent greater changes in the *D. labrax* experiment. Although this discrepancy can be partly attributed to the different rearing systems used, it should also be noted that *D. labrax* were monitored over a longer period (from 30 to 380 g) compared to *S. aurata* (from 220 to 352 g) [11, 15, 34, 38, 48–50]. The observed variation *in S. aurata* may be partly attributed to changes in stocking density due to fish growth across sampling times, despite the stocking density being considered low for this species [42, 51]. It is also worth mentioning that the feed changed from T0 to T1 and T2, but both feeds used were commercial ones adapted to the nutritional needs of the species, suggesting that this should not significantly impact the levels and contribution of globulin fractions (overall stable over the experiment duration, see Fig. 3B). Further specific studies will be needed to disentangle the various environmental effects on the different globulin contributions to total protein levels in these two species.

In *D. labrax*, we showed that the fraction of beta1 globulins contributed the most to the total protein content (34.16%), followed by albumins and alpha2 globulins (18.24 and 16.41% respectively), and this pattern was consistent across the three sampling times. Both the pre-albumin and alpha1 fractions contributed the least to the total protein content in *D. labrax* (5.49 and 7.71%, respectively). In contrast, in *S. aurata*, the albumin fraction contributed the most to the total protein content (23.39%), followed by beta1 and alpha2 globulins (19.71 and 19.15%, respectively). The gamma and alpha1 fractions contributed the least to the total protein content *in S. aurata* (5.34 and 8.63%, respectively). Interestingly, the three most contributing globulins are the same in both species studied, while only alpha1 was among the less contributing fractions in both species. High CVs were observed for the contribution of the pre-albumin fraction (64.37% and 27.30% for *D. labrax and S. aurata*, respectively), suggesting that the contribution of the pre-albumin fraction may vary significantly in healthy individuals of these species. Albumin is typically the most -or one of the most- contributing protein fraction to the total protein concentration due to its roles in transporting functions, as well as in blood volume regulation [11, 15]. Albumins have been reported to be affected by various factors in fishes (e.g., species features, season, stage of gonad maturity, temperature) [11, 15, 49], resulting in potentially, significant differences at the individual level. A prominent contribution of albumins is sometimes observed in both *D. labrax* and *S. aurata*, where albumin is consistently present in blood proteins at high percentages (e.g., [25, 27, 34, 50]). The high contribution of the albumin fraction can be a relevant indicator of health alteration in these species, particularly when fish are exposed to environmental variations, variable nutritional status or stressful conditions (e.g. [22, 34, 39, 52]). In contrast to the consistently high CV for the pre-albumin fraction in both species, low CVs were observed for the alpha2 and beta1 globulin fractions (alpha2: 12.59% and 17.73%; beta1: 16.02% and 18.17% for *D. labrax and S. aurata* respectively), suggesting that these globulins are more consistent contributors in healthy individuals. As previously mentioned, there are only a few studies that have measured all specific globulin fractions (i.e. alpha1 and 2 and beta1 and 2) for *S. aurata* and *D. labrax*, and results could differ from what we observed here [27, 34, 50]. For instance, [27] showed that in *D. labrax*, while both the albumin and alpha2 fractions were identified as major contributors to the total protein content, the contribution of beta globulins was low and gamma globulin was high, which was generally similar to the findings for albumin and alpha2 fractions, unlike what we found in this study. Similarly to our study, for *S. aurata*, [22] showed that alpha2 and beta1 globulin fractions had an overall similar contribution to the total protein content. Overall, since all fish in this study were healthy, we provide useful baseline information for future health assessment studies and highlight the need for species-specific investigations.

Finally, some correlations were observed between plasma concentrations of pre-albumin with alpha2, albumin with gamma, alpha1 with alpha2 and beta1, alpha2 with beta2, beta1 with beta2 and gamma, and beta2 with gamma in both species. These findings suggest strong inter-correlations between these protein concentrations in fishes, but further validation in additional species is necessary. Overall, in both species, albumins and globulins, especially within the alpha and beta fractions, exhibited significant inter-correlations. Likewise, strong inter-correlations were observed among globulins from the alpha, beta and gamma fractions. This was expected due to the similar biological functions of the correlated globulins, supporting the usefulness of analysing albumins, globulins and A/G ratio for fish health assessment [11, 14–16]. It is worth mentioning that, as observed in the present study and elsewhere [22, 32, 34], variations may occur within the alpha or beta fractions without affecting the other fraction (e.g., effect on alpha2 but not on alpha1). This underscores the importance of investigating all fractions to gain more specific insights into health effects.

### Size effect

In addition to environmental drivers, serum biochemical values can also be influenced by fish individual features, such as growth, sex, age and sexual maturation (e.g., [53–57]). Here, we only investigated the effect of mass.

The relationship between total protein content and body mass is species-specific. In this study, the overall increase in plasma total protein concentration over the three sampling times suggested that the total serum protein content was affected by fish body mass, regardless of the species investigated, in agreement with previous studies (e.g., [25, 58]). Correlations between weigh of *S. aurata* and *D. labrax* and total serum protein were previously demonstrated [59]. However, Fazio et al. showed a positive relationship for *D.* labrax, in agreement with this study, but a negative relationship for *S. aurata*, contrary to our findings here [59]. This negative relationship was explained by mobilization of tissue protein during growth, where amino acids would be used to synthesize new plasma proteins [59]. Further studies are necessary to explain these correlation differences. It is worth noting that the relationship between total serum protein content and weight is more pronounced in sea bream, possibly due to the relatively stable environmental conditions [11], as mentioned previously. When examining aquaculture species, it was found that total serum protein content is correlated with the number of white blood cells [10], a factor known to be associated with body mass [10, 60]. Additionally, the concentration of total protein in blood is known to be related to the specific growth rate in fishes [10], contributing to observed relationships between total serum protein concentration and body mass.

### Perspectives

Different environmental factors (e.g., temperature, season, salinity, stocking density) varied across different sampling times, as did individual features (life stage, age, body mass) in the two experiments. This complexity makes it challenging to disentangle the specific effects of environmental and intrinsic features. Nevertheless, the results of this study are important because they provide valuable data on the variation in concentrations and contributions of different fractions of total protein content in healthy individuals of two commercially important fish species, for which limited information is available. Specific control of environmental variations over time (e.g., focusing solely on water temperature variation) would be necessary to investigate the specific effects of environmental parameters and/or individual features for these two species. Additionally, in future studies, it would be valuable to compare the composition of globulin fractions in physiologically healthy fish with those in non-healthy fish, i.e. carriers of different diseases and/or fish experiencing stressful conditions. This will allow to specifically link variations in globulin concentration with specific diseases and use different globulins as relevant markers of disease in the context of health and welfare monitoring in aquaculture environments.

### Electronic supplementary material

Below is the link to the electronic supplementary material.

Supplementary Material 1

## Data Availability

The data that support the findings of this study are available from the corresponding author upon request.
