# Supplementary material for: Comparative analysis of blood protein fractions in two mediterranean farmed fish: Dicentrarchus labrax and Sparus aurata
Source: BMC Vet Res. 2024 Jul 18;20:322. doi: 10.1186/s12917-024-04182-w (PMC11256508; doi:10.1186/s12917-024-04182-w)
Supplement: Supplementary file 1 — Supplementary Material 1 [file 12917_2024_4182_MOESM1_ESM.docx]

**Supplementary material**

**Comparative analysis of blood protein fractions in two Mediterranean farmed fish: *Dicentrarchus labrax* and *Sparus aurata***

Sébastien Alfonso^a,b^*, Eleonora Fiocchi^c^, Lola Toomey^a^, Marilena Boscarato^c^, Amedeo Manfrin^c^, Arkadios Dimitroglou^d^, Leonidas Papaharisis^e^, Eleonora Passabi^f^, Annalisa Stefani^f^, Giuseppe Lembo^a^, Pierluigi Carbonara^a^

^a^ Fondazione COISPA ETS, Bari, Italy

^b^ Université Côte d'Azur, CNRS, ECOSEAS, Nice, France

^c^ National Reference Laboratory for Fish, Mollusc and Crustacean Diseases, Istituto Zooprofilattico Sperimentale delle Venezie, Legnaro, Italy

^d^ Department of Animal Science, Laboratory of Applied Hydrobiology, Agricultural University of Athens, Athens, Greece

^e^ Department of Research and Development, AVRAMAR S.A., Paiania, Greece

^f^ Laboratory Medicine Service, Istituto Zooprofilattico Sperimentale delle Venezie, Legnaro, Italy

*Corresponding author

Phone: +390805433596; email: [sebastien.alfonso1@gmail.com](mailto:sebastien.alfonso1@gmail.com)

**Supplementary Figures**

**Fig. S1**. Variations in temperature (°C; in blue) and oxygen concentration (g/L; in orange) in sea cages during the *D. labrax* experiment. Sampling times T1 and T2 are indicated in the figure.

**Fig. S2**. Variations (mean ± SD) in temperature (°C; in blue) and oxygen concentration (g/L; in orange) in tanks during the *S. aurata* experiment. Sampling times T0, T1 and T2 are indicated in the figure. Data are presented as mean ± SD (n=2 tanks).

**Supplementary Tables**

**Table S1**. Formulation and proximate composition of the feed used for the *D. labrax* experiment (% as fed).

| **Ingredients** | **Composition (% as fed)** |
| --- | --- |
| Fish meal | 20.00 |
| Soya Protein Concentrate | 4.80 |
| Soya bean meal | 19.50 |
| Fermented Soya | 5.70 |
| Sunflower meal | 7.20 |
| Corn Gluten | 18.80 |
| Wheat | 8.20 |
| Fish oil | 4.10 |
| Salmon oil | 10.30 |
| Monocalcium phosphate | 0.40 |
| Vitamins and Minerals | 0.70 |
| Methionine | 0.10 |
| Lysine | 0.20 |

**Table S2**. Formulation and proximate composition of the feed used for the *S. aurata* experiment (% as fed).

| **Ingredients** | **Composition (% as fed)** |
| --- | --- |
| Fish meal | 25 |
| Corn gluten meal | 22 |
| Soy Protein Concentrate | 5 |
| Soybean meal | 11 |
| Fish oil | 12 |
| Sunflower meal | 12 |
| Wheat | 10 |
| Premix | 3 |

**Table S3**. Zootechnical data for both experiments (*D. labrax* and *S. aurata*). Specific growth rate (SGR, %/day), feed conversion ratio (FCR) and mortality (n and %) over the experiment. Fish mass (g) is presented for each sampling time (T0, T1 and T2) for sampled fish (see details in Fig. 1 for sample size) and the entire fish populations (i.e. all tanks). Data are presented as mean ± SD.

| **Variable** | ***D. labrax*** | ***S. aurata*** |
| --- | --- | --- |
| SGR (%/day) | 0.69 ± 0.01 | 0.46 ± 0.20 |
| FCR | 2.06 ± 0.09 | 2.28 ± 2.1 |
| Mortality (n) | 483 ± 29 | 3 ± 1 |
| Mortality (%) | 22.03 ± 1.30 | 3.57 ± 1.01 |
| Mass at T0 (g) - all | 40.9 ± 0 | 219.7 ± 50.0 |
| Mass at T0 (g) - sampled | 29.7 ± 4.2 | 196.5 ± 18.11 |
| Mass at T1 (g) - all | 337.7 ± 2.1 | 301.1 ± 59.0 |
| Mass at T1 (g) - sampled | 318.3 ± 140.3 | 295.8 ± 60.3 |
| Mass at T2 (g) - all | 474.8 ± 9.6 | 359.7 ± 76.9 |
| Mass at T2 (g) - sampled | 379.5 ± 148.6 | 352.1 ± 81.8 |
